# Neuroprotection by Flaxseed Oil in a Model of Hippocampal Injury Induced by Trimethyltin Involves Purinergic System Modulation

**DOI:** 10.3390/ijms262110283

**Published:** 2025-10-22

**Authors:** Nataša Mitrović, Marina Zarić Kontić, Ivana Grković

**Affiliations:** Department of Molecular Biology and Endocrinology, VINČA Institute of Nuclear Sciences-National Institute of the Republic of Serbia, University of Belgrade, 11000 Belgrade, Serbia; natasa@vin.bg.ac.rs (N.M.); marinazaric@vin.bg.ac.rs (M.Z.K.)

**Keywords:** hippocampus, trimethyltin, flaxseed oil, antioxidative enzymes, purinergic receptors, NTPDase1-eN, adenosine system

## Abstract

A large body of evidence suggests that flaxseed oil (FSO), one of the richest sources of essential omega-3 fatty acids, has neuroprotective properties. Purinergic signaling plays a crucial role in pathophysiological processes in the nervous system. There is a lack of evidence regarding the effects of FSO on the purinergic system under both physiological and neurotoxic conditions. Here we report the effects of dietary FSO consumption in a rat model of trimethyltin (TMT) intoxication. Exposure to TMT selectively induces hippocampal neuronal damage and glial reactivation associated with oxidative stress and neuroinflammation, causing severe behavioral impairments. When administered orally (1 mL/kg) before and during TMT intoxication (single dose 8 mg/kg, i.p.) to female Wistar rats, FSO effectively prevented the behavioral disturbances induced by TMT. FSO selectively increased CAT-mRNA level in both healthy and TMT-intoxicated animals, while preventing TMT-induced upregulation of Nrf2, NF-κB, and GPx1 without affecting SOD2 or Gsr-mRNA levels. FSO prevented microgliosis, microglial NTPDase1-eN upregulation, and the increase in purinergic receptors involved in microglial reactivity. Pretreatment with FSO in TMT-intoxicated rats maintained the activity and expression of NTPDase1 at control level, while the activity and expression of eN and ADA were increased. FSO upregulated eN, A_1_R, A_2B_R, A_3_R, ADA, and NGF, while downregulating NTPDase1, A_2A_R, and ENT1 in TMT-intoxicated rats. This suggests complex modulation of purinergic signaling, particularly the adenosine system. These findings may contribute to a better understanding of the effects of FSO, highlighting the impact of the dietary intake of this oil on the brain.

## 1. Introduction

Plant-based food is rich in bioactive compounds that exert various beneficial effects in the body [1,2]. Among them, flaxseed (*Linum usitatissimum*) is one of the richest plant source of omega-3 and omega-6 polyunsaturated fatty acids such as α -linolenic acid (ALA, C18:3) and linoleic acid (LA, C18:2), respectively, as well as essential soluble dietary fibers, high-quality proteins, lignans, and numerous phytochemicals [1,3]. Flaxseed health benefits, including cardioprotective, anti-diabetic, anti-inflammatory, immuno-modulatory, and anticancer effects, are mainly attributed to its constituents of omega-3 polyunsaturated fatty acids (n-3 PUFA) and the phenolic lignans [1]. As a powerful food with anti-inflammatory and antioxidant properties [4,5], the flaxseed-derived product flaxseed oil (FSO) is a promising dietary intervention for the prevention of neurodegeneration and promotion of brain health [6,7,8,9]. n-3 PUFA from FSO have similar functions in the brain as in other tissues, such as production of less inflammatory and less aggregatory eicosanoids, which may preserve or enhance brain function [10]. They may improve the control of neuronal membrane excitability [11], enhancing its fluidity, permeability, and synaptic plasticity [12]. n-3 PUFA from FSO also increase cerebral blood flow and decrease ischemic damage to neurons, thus improving neuronal survival and restoring brain function [3,13]. FSO supplementation strengthens the antioxidant defense system and mitigates oxidative stress by augmenting antioxidant capacity and reducing lipid peroxidation [3,7,14]. The transcriptional regulator of antioxidative enzymes, nuclear factor erythroid 2-related factor 2 (Nrf2) and nuclear factor kappa-light-chain-enhancer of activated B cells (NF-κB) pathways co-regulate cellular responses to oxidative stress and inflammation as the first defense mechanism [15,16]. However, the effect of FSO diet on the gene expression of primary defense enzymes against oxidative stress such as superoxide dismutase (Sod), glutathione peroxidase 1 (Gpx1), glutathione reductase (Gsr), and catalase (Cat) is still poorly investigated in vivo.

FSO is reported to influence the production and activity of the monoamine neurotransmitter system [7,17]. Another central modulator of neuronal and glial function is the purinergic signaling system, which controls processes such as synaptic plasticity, neuroinflammation, neurodevelopment, and energy homeostasis [18]. This complex system involves receptors, transporters, and ectonucleotidases (enzymes that maintain extracellular ATP/adenosine balance), thereby affecting glial activation and neurotransmission. Under physiological conditions, tonic ATP release and activation of P2 receptors regulate brain function, while excessive ATP release during pathology promotes cytokine secretion from astrocytes and microglial migration [18,19,20]. Ecto-enzymes such as ecto-nucleoside triphosphate diphosphohydrolase (NTPDase1/CD39) and ecto-5’-nucleotidase (eN/CD73) hydrolyze ATP/ADP to AMP and adenosine, maintaining nucleotide signaling homeostasis [21,22]. Adenosine, a potent neuromodulator, acts via P1 receptors (A_1_R, A_2A_R, A_2B_R, A_3_R) to regulate neurotransmission, exert neuroprotective and anticonvulsive effects by activating inhibitory and facilitatory A_1_R and A_2A_R [23,24,25], and support neuronal survival through NGF release [26,27]. It is further metabolized by adenosine deaminase (ADA) or transported via directly into the cells by equilibrative nucleoside transporters (ENTs), with ENT1 showing the highest affinity for adenosine [25,28,29]. Since dysregulation of purinergic/adenosine signaling is linked to epilepsy, neurodegeneration, and psychiatric disorders [30], its modulation represents a promising therapeutic strategy. However, the effects of FSO on the purinergic/adenosine system remain unexplored in both health and disease.

To investigate effects of FSO, we used the trimethyltin (TMT) model of neurodegeneration, which is considered as a valid tool for the in vivo modeling of Alzheimer’s disease and temporal lobe epilepsy (TLE) [31,32,33]. TMT is an intermediate by-product of organotin substances used in industrial and agricultural settings that can cross the blood–brain barrier and induce neuronal damage in several brain regions, particularly limbic structures [34,35,36,37]. TMT intoxication initiates a variety of neurological symptoms, including ataxia, disorientation, aggressive behavior, seizures, sensory neuropathy, and memory loss in humans and in rodents as well. Several underlying mechanisms include glutamate excitotoxicity, intracellular calcium overload, neuroinflammation, oxidative stress, mitochondrial dysfunction, and alteration in gene expression, resulting in cell death [38,39,40,41]. TMT triggers an immediate astrocyte response toward the proinflammatory phenotype which contributes to the acceleration of neuroinflammation and neuronal damage, and reactivation of resident microglial cells [35,42,43]. In accordance, purinergic signaling is affected during TMT-induced neurodegeneration. Microglial P2 receptors, such as P2Y_12_, P2X_4_R, and P2Y_6_R, are upregulated at the early stage of TMT-induced neurodegeneration as well as NTPDase1/eN [42]. In addition, selective switch of adenosine receptors from neurons to astrocytes at the early stage of TMT-induced neurodegeneration have been reported [42].

Our previous work has shown that FSO supplementation prevented TMT-induced hippocampal cell death, and reduced astrocytic activation and their polarization towards a proinflammatory/neurotoxic phenotype, thus promoting neuroprotection [39]. Given the demonstrated importance of antioxidant protection and purinergic/adenosine signaling in the mechanisms of neurodegeneration and neuroprotection and their functional interplay in regulating cellular redox balance, inflammation, and neurotransmission, we sought to further clarify how FSO exerts its effects. Given that FSO can modulate the expression of genes involved in antioxidant and anti-inflammatory pathways in peripheral tissues [3,4,5], we investigated its impact on the expression of key antioxidant enzymes in the brain. Since there are no data on how FSO per se or in the context of a neurodegenerative insult affects purinergic signaling, our study focused on how FSO impacts the expression of components of the purinergic/adenosine system at the early stages of TMT-induced neurodegeneration.

## 2. Results

### 2.1. FSO Pretreatment Alleviated TMT-Induced Behavior Symptoms

**Figure 1:**
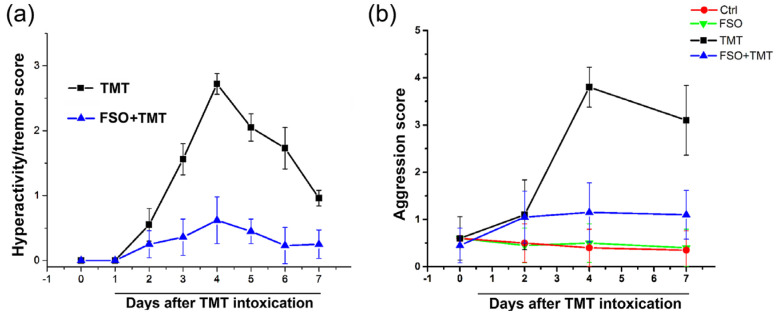
Assessment of hyperactivity/tremor and aggression. (**a**) Changes in tremor severity score post-intoxication. Hyperactivity/tremor signs were scored according to a 0–3 scale (0—without symptoms; 1—hyperactivity and hyper-responsiveness, 2—mild tremor with normal motor activity; 3—systemic tremor). (**b**) Changes in aggression severity score post-intoxication. Aggression signs were scored according to a 0–4 scale (0—remains calm when approached and grasped; 1—shies from hand when grasped; 2—avoids hand by running, struggles when captured, or both; 3—leaps to avoid capture and struggles vigorously when captured; 4—leaps, struggles, and bites when captured. The data are reported as the mean ± SEM (*n* = 15 animals/group).

The behavioral score was assessed over a seven-day period after TMT intoxication, as previously reported [41,43]. Hyperactivity/tremor score exhibited a bell-shaped curve that peaked on day 4 and decreased on day 7 (Figure 1a), which is a time-point defined as an early stage of TMT-induced neurodegeneration [42,43]. The FSO-treated animals showed milder behavioral symptoms, which resulted in a flattened behavioral score curve. Animals also developed aggressiveness [41,44]. The aggression score also peaked on day 4 after TMT intoxication, and remained elevated till 7 days post-TMT (Figure 1b). In FSO-treated TMT-intoxicated animals, aggressiveness is also reduced to a normal level.

### 2.2. Effect of FSO on Antioxidant Genes Expression in the Hippocampus

**Figure 2:**
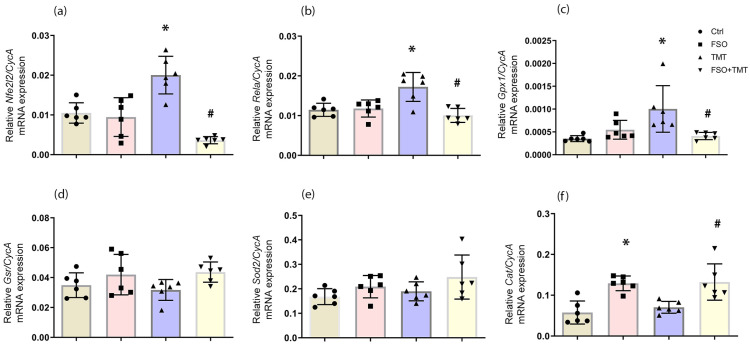
Effect of FSO on the mRNA levels of (**a**) *Nfe2l2*, (**b**) *Rela* (**c**) Gpx1, (**d**) Gsr, (**e**) Sod2, (**f**) Cat in the hippocampus of control (Ctrl), flaxseed oil (FSO), trimethyltin (TMT), and flaxseed oil-treated TMT-intoxicated (FSO + TMT) animals. Bars represent mean mRNA expression of target gene relative to CycA ± SD (*n* = 6 animals/group). Significance shown inside the graphs: * *p* < 0.05 or less relative to Ctrl; # *p* < 0.05 or less relative to TMT.

First, we assessed how FSO alters expressions of genes involved in the antioxidant defense system in the hippocampus of healthy and TMT-intoxicated animals. There were no changes in mRNA levels in the FSO group—Nrf2 (*Nfe2l2*) and p65 subunit of NF-κB (*Rela*)—compared to the control group (Figure 2a,b). qPCR analyzes showed upregulation of both Nrf2- and NF-κB-mRNA levels in the TMT group (*p* < 0.01 for both) compared to the control group. In FSO + TMT groups, FSO prevented Nrf2- and NF-κB-mRNA upregulation and decreased them compared to TMT (*p* < 0.001 for both). FSO pretreatment did not affect Sod2-, Gpx1-, and Gsr-mRNA levels (Figure 2c–e). In the TMT group, only Gpx1-mRNA level increased (*p* < 0.01), and FSO pretreatment prevented this alteration (*p* < 0.01, compared to TMT) (Figure 2c). FSO significantly increased Cat-mRNA level (*p* < 0.01) compared to the control group (Figure 2f). Although TMT did not alter Cat-mRNA, its level increased in FSO + TMT group (*p* < 0.01) compared with TMT (Figure 2f). The results of two-way ANOVA for qPCR of the analyzed genes are presented in Supplementary Material Table S1.

### 2.3. FSO Pretreatment Modulates NTPDase1/eN and Expression of Purinergic Receptors Involved in TMT-Induced Microgliosis

**Figure 3:**
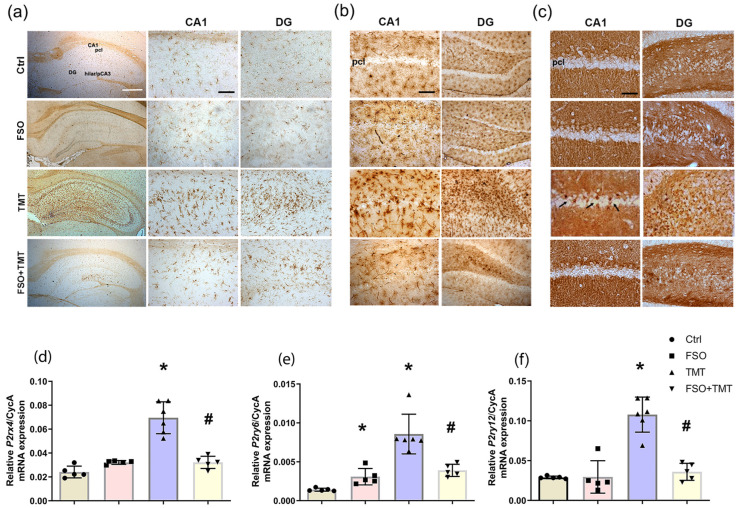
Effects of FSO pretreatment on TMT-induced microgliosis and NTPDase1-eN enzyme localization in the hippocampus. Representative (**a**) Iba1-immunoreactivity, ectonucleotidase histochemistry in the presence of (**b**) ADP and (**c**) AMP as substrate and corresponding enlarged CA1/DG subregions of control (Ctrl), flaxseed oil (FSO), trimethyltin (TMT), and flaxseed oil-treated TMT-intoxicated (FSO + TMT) animals. Black arrows indicate AMPase-labeled individual round-shaped cells in the pcl of CA1 Scale bar applicable to lower magnifications—500 μm and 50 μm applicable to higher magnifications. DG—dentate gyrus; pcl—pyramidal cell layer; hilar/pCA3—hilus of DG and proximal part of CA3 region. Effects of FSO pretreatment on the abundances of transcripts coding for (**d**) P2X_4_R, (**e**) P2Y_6_R, (**f**) P2Y_12_R were assessed by RT-qPCR. Bars represent mean mRNA expression of target gene relative to CycA ± SD (*n* = 5−6 animals/group). Significance shown inside the graphs: * *p* < 0.05 or less relative to Ctrl; # *p* < 0.05 or less relative to TMT.

As reported [42], microglial activation induced by TMT is manifested as a robust increase in overall intensity of Iba1- immunoreactivity (*ir*), delineating cells with mixed morphology that gradually populated hippocampus seven days after TMT intoxication (Figure 3a). FSO pretreatment prevented the overall increase in Iba1-*ir* and change in their morphology observed after TMT intoxication, although Iba1^+^ cells with enlarged cell bodies were occasionally observed in the DG (Figure 3a).

Microglia are cells that primarily express NTPDase1 [42,45], which is the main enzyme for the conversion of extracellular ATP and ADP to AMP [21]. The localization of NTPDase1 was determined by enzyme histochemistry using ADP as a substrate, that primarily labels microglial cells and also matches Iba1-*ir* [42,45]. In the FSO group, we could not detect any change in the spatial distribution of ADPase localization compared to Ctrl. At the early stage of TMT-induced neurodegeneration, the lead phosphate deposits stained reactive microglia that covered the strata, but also entered the neuronal layers (Figure 3b). FSO pretreatment of TMT-intoxicated animals attenuated the overall increase in ADPase activity on microglial cells, but as for Iba1-*ir*, occasional ADPase-labeled microglia with enlarged cell bodies were observed in DG.

The localization of eN enzyme activity in the hippocampus was determined by enzyme histochemistry using AMP as a substrate, which labels synaptic layers, while neuronal cell layers remained unstained in healthy hippocampi and completely corresponded to the eN-*ir* [42,45]. In the FSO group, we could not detect any change in spatial distribution of AMPase localization compared to Ctrl (Figure 3c). In the TMT group, AMPase-labeled individual round-shaped elements were present in the pyramidal cell layer of CA1 and neuronal layers of DG, as reported [42]. Notably, these AMPase-labeled round cells were absent in the FSO-treated TMT-intoxicated animals (Figure 3c). 

Regarding ATP/ADP-sensitive P2 receptors expressed by microglia, a significant increase in the relative abundance of P2X_4_R-, P2Y_6_R-, and P2Y_12_R-mRNA levels was observed at the early stage of TMT-induced neurodegeneration (*p* < 0.0001, *p*  <  0.0001, *p*  <  0.0001, respectively) compared to Ctrl (Figure 3d–f), as previously reported [42]. Although FSO alone had no effects on these receptors, pretreatment of TMT-intoxicated animals with FSO completely prevented upregulation of P2X_4_R-, P2Y_6_R-, and P2Y_12_-mRNA levels (*p* <  0.001, *p* <  0.001, *p* <  0.001, respectively) compared to TMT (Figure 3d–f). Two-way ANOVA results for qPCR analysis of the purinergic receptors examined are shown in Table S1 in the Supplementary Material.

### 2.4. FSO Modulates Activity and Expressions of NTPDase1 and eN

**Figure 4:**
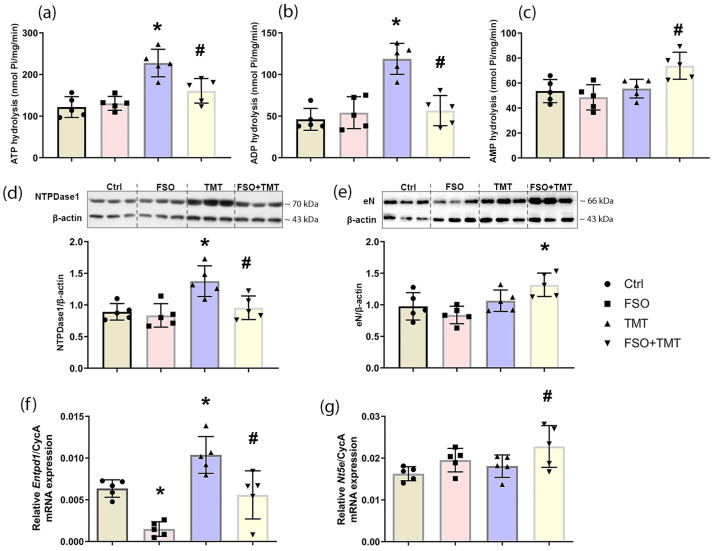
Effects of FSO pretreatment on functional activity and expression of NTPDase1-eN enzymes in the hippocampal region of control (Ctrl), flaxseed oil (FSO), trimethyltin (TMT), and flaxseed oil-treated TMT-intoxicated (FSO + TMT) animals. (**a**,**b**) Levels of NTPDase1 activity in the presence of ATP, ADP, and (**c**) eN activity in the presence of AMP, assessed by determining the level of free inorganic phosphate (Pi) in the reaction medium. Bars represent mean hydrolysis activity (nmol Pi/mg/min) ± SD in the membrane preparations from five animals/group performed in triplicate. Representative support membranes and relative protein expressions of (**d**) NTPDase1 and (**e**) eN. Bars represent mean protein abundance relative to β-actin ± SD, from five separate determinations performed in triplicate (*n* = 5 animals/group). The abundances of transcripts coding for (**f**) NTPDase1 and (**g**) eN were assessed by RT-qPCR. Bars represent mean mRNA expression of target gene relative to CycA ± SD (*n* = 5 animals/group). Significance shown inside the graphs: * *p* < 0.05 or less relative to Ctrl; # *p* < 0.05 or less relative to TMT.

To assess functional activity of ectonucleotidases in the hippocampus, we further investigated whether FSO pretreatment alters the activity and expression of the NTPDase1-eN enzyme chain in both healthy and TMT-intoxicated animals. The levels of ATP and ADP hydrolysis in the hippocampal membranes were not altered by FSO alone (Figure 4a,b). TMT induced significant increase in ATP and ADP hydrolysis (*p* < 0.001 for both) in respect to Ctrl, which is prevented by FSO pretreatment of TMT-intoxicated animals (*p* < 0.001 for both) compared to TMT. Consistent with alterations in NTPDase1 activity, immunoblot analysis confirmed increased protein abundance of NTPDase1 in TMT-intoxicated animals (*p* < 0.01 compared to Ctrl), and prevention of this upregulation in the TMT + FSO group (*p* < 0.05 compared to TMT) (Figure 4d). At the mRNA level, TMT significantly upregulated NTPDase1 transcripts (*p* < 0.05) in respect to Ctrl (Figure 4f), as we showed previously [42]. A significant decrease in NTPDase1-mRNA level was observed in the FSO group (*p* < 0.01) compared to Ctrl, indicating the direct effects of FSO pretreatment on NTPDase1 gene expression. FSO pretreatment of TMT-intoxicated animals also prevented upregulation of NTPDase1 in the hippocampus and significantly decreased the relative abundance of NTPDase1-mRNA (*p* < 0.01) compared to TMT. Two-way ANOVA results for NTPDase1 enzyme assay, immunoblot, and qPCR analysis are shown in Tables S1–S3 in the Supplementary Materials.

Ecto-5′-nucleotidase (eN) is the main adenosine-producing enzyme [21]. Results showed that FSO and TMT did not alter AMP hydrolysis, which reflects eN activity in the hippocampal membranes (Figure 4c); while in FSO-pretreated TMT-intoxicated animals, it was slightly increased compared to the TMT and FSO group (*p* < 0.05 and *p* < 0.01, respectively). Consistently, eN protein abundance is increased only in the FSO + TMT group compared to FSO alone (*p* < 0.05, Figure 4e). The qPCR analysis showed that neither FSO alone nor TMT intoxication had effects on eN-mRNA levels, whereas a slight increase was observed in the FSO-treated TMT-intoxicated group compared to Ctrl and TMT (*p* < 0.05 for both, Figure 4g). Two-way ANOVA results for eN enzyme assay, immunoblot, and qPCR analysis are shown in Tables S1–S3 in the Supplementary Materials.

### 2.5. FSO Modulates Expression of Adenosine Receptors

**Figure 5:**
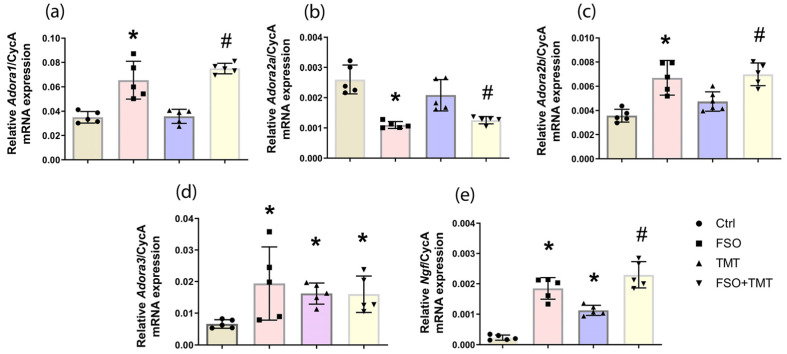
FSO pretreatment modulates adenosine receptors. The abundances of transcripts coding for (**a**) A_1_R, (**b**) A_2A_R, (**c**) A_2B_R, (**d**) A_3_R, and (**e**) NGF in the hippocampal region of control (Ctrl), flaxseed oil (FSO), trimethyltin (TMT), and flaxseed oil-treated TMT-intoxicated (FSO + TMT) animals were assessed by RT-qPCR. Bars represent mean mRNA expression of target gene relative to CycA ± SD (n = 5 per group). Significance shown inside the graphs: * *p* < 0.05 or less relative to Ctrl; # *p* < 0.05 or less relative to TMT.

Signaling actions of adenosine in the brain are mainly mediated via high-affinity inhibitory A_1_R and excitatory A_2A_R receptors, which are differentially involved in pathophysiological processes [25,46]. The qPCR analysis showed that FSO alone induced significant increase in A_1_R-mRNA abundance (*p* < 0.001), while it induced decrease in A_2A_R-mRNA (*p* < 0.001) compared to Ctrl (Figure 5a,b). TMT had no effects on the total amount of A_1_R- or A_2A_R-mRNA in the hippocampus compared to Ctrl as reported previously [42], while FSO pretreatment of TMT-intoxicated animals significantly increased A_1_R-mRNA (*p* < 0.001), and decreased A_2A_R-mRNA (*p* < 0.05) compared to TMT. Moreover, both FSO alone and FSO + TMT induced an increase in low-affinity A_2B_R-mRNA (*p* < 0.001 compared to Ctrl and *p*  <  0.05 compared to TMT, respectively) (Figure 5c). Finally, FSO pretreatment induced a slight increase in A_3_R-mRNA levels (*p*  <  0.05 in both FSO and FSO + TMT groups), with a similar effect observed following TMT intoxication (*p* < 0.05 vs. Ctrl) (Figure 5d). It is known that A_1_R can cause NGF release [26,27]. The qPCR demonstrated that consumption of FSO induced a significant upregulation of NGF-mRNA compared to Ctrl (*p* < 0.001, Figure 5e). In addition, upregulation of NGF-mRNA was observed in the TMT group (*p* < 0.01) compared to Ctrl, and further increased in the FSO-treated TMT group compared to TMT (*p* < 0.001). The results of two-way ANOVA for qPCR analysis of the analyzed genes are shown in Supplementary Material Table S1.

### 2.6. FSO Modulates Expression of ADA and ENT1

**Figure 6:**
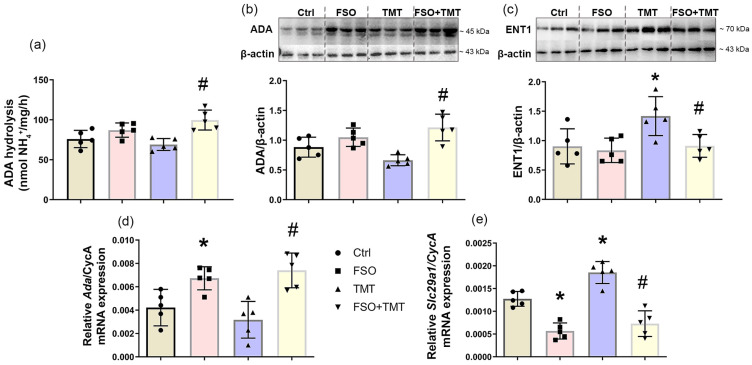
Effects of FSO pretreatment on functional activity and expression of ADA and ENT1 in the hippocampal region of control (Ctrl), flaxseed oil (FSO), trimethyltin (TMT), and flaxseed oil-treated TMT-intoxicated (FSO + TMT) animals. (**a**) Levels of ADA activity. Bars show mean activity (nmol NH_4_^+^/mg/h) ± SD from individual sample preparations performed in triplicate from five animals per group. Representative support membranes and relative protein abundance of (**b**) ADA and (**c**) ENT1. Bars represent mean protein abundance relative to β-actin ± SD, from five separate determinations performed in triplicate (*n* = 5 animals/group). The abundances of transcripts coding for (**d**) ADA and (**e**) ENT1 (*Slc29a1*) assessed by RT-qPCR. Bars represent mean mRNA expression of target gene relative to CycA ± SD (*n* = 5 animals/group). Significance shown inside the graphs: * *p* < 0.05 or less relative to Ctrl; # *p* < 0.05 or less relative to TMT.

To characterize the regulation of adenosine metabolism, we examined the activity and expression of adenosine-metabolizing enzyme ADA and the expression of major adenosine transporter, ENT1 (Figure 6). FSO alone did not affect ADA activity and protein abundance but increased its mRNA level (*p* < 0.05) compared to Ctrl. Although there were no changes in ADA activity, protein abundance, and mRNA level in TMT group compared to Ctrl, the results indicated an increase in ADA activity, relative protein abundance, and mRNA level in the FSO + TMT group compared to TMT (*p* < 0.01, Figure 6a,b,d). Immunoblot analysis showed that TMT increased relative protein abundance of ENT1 (*p* < 0.05) compared to Ctrl, while FSO treatment of TMT-intoxicated animals prevented this increase (*p* < 0.05 compared to TMT) (Figure 6c). The qPCR analyzes showed that TMT significantly increased ENT1-mRNA level (*p* < 0.001) compared to Ctrl (Figure 6e). FSO alone as well as FSO treatment of TMT-intoxicated animals induced significant decrease in ENT1-mRNA levels (*p* < 0.001 and *p* < 0.001, respectively) compared to both Ctrl and TMT (Figure 6e). The results of two-way ANOVA for assay, immunoblot, and qPCR of the analyzed ADA and ENT1 are presented in Supplementary Materials Tables S1–S3.

## 3. Discussion

Different dietary interventions exert a profound influence on human health and disease progression. Bioactive compounds from flaxseed/FSO maintain brain health through their anti-inflammatory, antioxidative, and modulatory properties [1,2,47,48]. These effects are associated with the presence of essential soluble dietary fibers, n-3 PUFAs, high-quality proteins, lignans, and various phytochemicals. Our previous work demonstrated that FSO consumption effectively attenuated cell death, preserved hippocampal neuronal integrity, and prevented proinflammatory polarization of astrocytes following TMT intoxication at both histological and molecular levels [39]. Additionally, these beneficial effects were accompanied by an increased intrahippocampal content of n-3 PUFAs. Here, we demonstrated that dietary FSO consumption prevented the occurrence of TMT-induced behavioral impairments characteristic of the early stage of TMT intoxication [35,36,43,44]. Specifically, FSO reduced the severity of symptoms such as hyper-excitability, hyper-responsiveness, and aggression, supporting its neuroprotective potential against TMT-induced toxicity.

Disruption of oxidative and antioxidative balance contributes to cognitive decline and behavioral issues such as seizures, aggression, and hyperactivity [36,37,41,49]. The altered oxidative milieu impacts redox-sensitive transcription factors like Nrf2 and NF-κB, which are key components of cellular responses to oxidative stress and inflammation [16,50,51]. Nrf2 can counteract NF-κB by upregulating antioxidant genes and suppressing inflammation, while NF-κB can affect Nrf2 activity. In line with this, our results showed a significant upregulation of Nrf2 and NF-κB mRNA in the hippocampus of TMT-intoxicated animals, likely as a compensatory response. Nrf2 upregulation correlated with seizure onset after TMT intoxication [35], aligning with previous findings in the epileptic tissue, and suggesting a neuroprotective attempt to restore redox balance under oxidative conditions [52]. The activities of pro- and antioxidative enzymes have been widely studied in TMT model, e.g., [41,49,53]. Among investigated antioxidant enzymes, only Gpx1 was upregulated at the early stage of TMT neurodegeneration, reflecting a defense mechanism against redox imbalance. It was reported that FSO supplementation might play a beneficial role in the reinforcement of the antioxidant defense system and amelioration of oxidative stress [4,5,14]. Thus, FSO prevented Nrf2 and NF-κB upregulation, indicating it may reduce oxidative burden upstream, rather than triggering stress-related transcription [3]. Effects of FSO supplementation on gene expression of antioxidative enzymes are poorly investigated in the brain. Prior studies showed that FSO increases Sod2, Cat, and Gpx gene expression in the liver of diabetic rats [4]. In our study, FSO selectively elevated Cat-mRNA in both healthy and TMT-intoxicated animals, and is an enzyme with a crucial role in protecting cells from the harmful effects of reactive oxygen species, which makes it a potential therapeutic target [54]. FSO also prevented TMT-induced GPx1 upregulation but without altering mitochondrial Sod2 or Gsr levels. These results suggest that FSO probably reinforces specific antioxidant defense and limits maladaptive stress responses. Such targeted modulation likely contributes to maintaining redox balance, thereby reducing tissue vulnerability to TMT-induced injury.

Another fundamental regulator of cellular homeostasis and neuron–glia communication is the purinergic signaling system, which involves extracellular adenosine and nucleotides (like ATP) that bind to cell-surface P1 and P2 receptors and regulate physiological and pathological processes [18]. As we have previously showed, the purinergic system is affected during TMT-induced neurodegeneration and involved in microglia reactivation [42]. FSO pretreatment of TMT-intoxicated animals prevented upregulation of NTPDase1 in microglia at the early stage of TMT-induced neurodegeneration and also prevented infiltration of eN-positive amoeboid cells in the hippocampal neuronal strata. Furthermore, FSO consumption prevented upregulation of mRNA levels of ATP- and ADP-sensitive purinergic receptors, P2Y_12_, P2X_4_R, and P2Y_6_R, which are responsible for the activation of microglial process extension, regulation of chemotaxis, and migration to the site of injury [20]. Without injury, there is no need for microglial migration, transformation, and overexpression of these enzymes and receptors.

When we consider the effects of FSO pretreatment on the functional activity and expression of NTPDase1 in the hippocampus both alone and in TMT-intoxicated animals, we can hypothesize that one of the mechanisms by which FSO exerts its beneficial effects is through the adaptive attenuation of NTPDase1 gene expression, thus maintaining relative protein level/activity in cell membranes. The absence of increased hydrolyzing activity of eN in TMT-intoxicated animals indicates AMP accumulation and insufficient production of adenosine. It is known that increasing adenosine levels plays an important role in preventing cell damage and dysfunction. The slight increase in eN expression and activity observed in the FSO + TMT group may therefore indicate a context-dependent modulatory effect of FSO under toxic conditions and might allow a more efficient ATP-derived adenosine formation. However, this assumption requires further clarification.

Adenosine is generally considered as a potent neuroprotective molecule, but it may exert pleiotropic actions depending on its functional coupling with a particular P1 receptor subtype [25,46]. A_1_R and A_2A_R exhibit a dynamic equilibrium, maintaining the inhibitory effects of the adenosine system due to the high expression of neuronal A_1_R in the hippocampus. Although the total mRNA levels of A_1_R and A_2A_R were not altered at the early stage of TMT-induced neurodegeneration, we previously found their upregulation on proinflammatory astrocytes in the injured area [42]. The shift in A_1_R/A_2A_R from neurons to astrocytes could favor excitotoxicity [55,56], and render neurons vulnerable to secondary effects of TMT, such as seizures [25,35]. The adenosine receptors are the most affected by FSO pretreatment. The potential of FSO to alter the expression of numerous genes has been found in peripheral tissues and cell cultures [3,4,5]. Bioactive components of FSO, especially n-3 PUFA and lignans, can influence receptor expression and downstream signaling by altering lipid raft integrity and intracellular second messenger systems [3]. Such membrane remodeling could consequently modulate adenosine receptor expression and function. Upregulation of A_1_R and the possible enhancement and preconditioning of A_1_R signaling by FSO could reduce glutamate release, prevent excitotoxic damage, and ameliorate neuronal hyperactivity and dysfunction [46]. In contrast to A_1_R, FSO pretreatment led to the attenuation of A_2A_R expression. Downregulation of A_2A_R conferred a robust tissue protection against brain damage and prevented synaptic dysfunction in various models of neurodegeneration/neuroinflammation [46,57,58,59]. Low-affinity A_2B_R are co-expressed with A_2A_R in most cases and their activation exerts anti-inflammatory effects by inhibiting vascular adhesion and migration of proinflammatory cells [60,61,62]. Upregulation of A_2B_R by FSO pretreatment could control A_2A_R and/or compensate for its decreased mRNA level. FSO could also stimulate A_2B_R expression as part of an adaptive response to improve brain energy metabolism, and together with increased A_1_R expression, they support synaptic stability and cognitive enhancement [61]. On the other hand, A_3_R mRNA level is increased after TMT intoxication, which is considered as an endogenous protective mechanism [63]. A_3_R interacts with P2Y_12_R to mediate microglial process extension and migration to the site of active neurodegeneration in response to ATP and adenosine [20,64,65]. The antioxidant properties of FSO may enhance the expression of widespread A_3_R, whereby its activation is linked to anti-inflammatory and anti-apoptotic pathways [63,65,66].

It is well known that NGF improves the plasticity of the brain, protects tissue from neuronal damage, and has anticonvulsive properties [67]. The parallel increase in both A_1_R and NGF expression by FSO may suggest a coordinated neuroprotective response, since the activation of A_1_R has been shown to induce NGF expression and stimulate its release [26,27]. This increase in NGF-mRNA level aligns with previous reports that FSO modulates the expression of neurotrophic factors. Specifically, FSO induces an increase in BDNF and GDNF levels following ischemic brain stroke, thus improving functional motor recovery [9]. In the context of TMT-induced neurotoxicity, FSO also elevated hippocampal BDNF expression [39], supporting its role in promoting neuronal resilience and functional recovery.

Finally, adenosine metabolism and transport are essential for maintaining extracellular adenosine levels, and their dysfunction and disruption can impact adenosine receptor signaling and brain function [24]. ADA converts adenosine to inosine, regulating extracellular adenosine levels [28]. It also fine-tunes adenosine signaling by allosterically modulating ARs to enhance receptor function and contributes to cell–cell communication [28,68,69]. While TMT intoxication did not alter ADA activity and expression, upregulation along with increased eN activity was observed in FSO-pretreated TMT-intoxicated animals, possibly preventing desensitization of the adenosine receptor. This suggests that FSO may enhance the degradation of adenosine, thereby maintaining a balanced extracellular adenosine environment.

The equilibrative nucleoside transporter ENT1 is the main regulator of extracellular adenosine in the brain, responsible for the concentration-dependent influx or efflux of adenosine into astrocytes and neurons [28,70]. TMT exposure led to an increase in ENT1 expression at the early stage of neurodegeneration, which is a time frame that coincides with the onset of seizures after TMT intoxication as well as with clearly visible cell damage [35,39,43]. This transporter is also upregulated under pathophysiological conditions such as ischemia and inflammation, in seizure animal models, and in patients with epilepsy, as well as in different neurodegenerative conditions [24,29,70,71]. These studies suggest that ENT1 plays a critical role in the control of immune responses, mitochondrial activity, and neurological functions. FSO consumption prevented increase in ENT1 protein abundance in the hippocampus under toxic conditions. It is well known that pharmacological inhibition or downregulation of ENT1 has antiepileptic effects in seizure models [70,72]. In our experimental setup, the mRNA level of ENT1 was significantly attenuated in both FSO groups, suggesting that FSO pretreatment leads to modulation of ENT1 gene expression in the rat hippocampus, thus contributing to the prevention of neurodegeneration.

Although this study suggests that FSO modulates the expression of components of the purinergic system and thus represents a potential strategy to prevent neurodegeneration, our study has some limitations. Changes in mRNA expression do not necessarily correlate directly with protein levels due to complex regulation and cell-specific, microenvironment-dependent expression. Adenosine receptors shift from neurons to glial cells under pathological conditions such as TMT intoxication [42], so the overall unchanged mRNA levels do not reflect their up- or downregulation in specific cell types. In addition, direct interactions between FSO and receptors have not been tested. It is known, for example, that n-3 and n-6 PUFAs themselves alter membrane fluidity and permeability, thereby changing the density of various receptors, including adenosine A_1_R in several brain regions [17,73]. Additionally, different fat types can increase or decrease receptor binding and may interact directly to alter their density levels in the membranes. By examining mRNA levels of adenosine receptors, we cannot speculate whether FSO binds directly to these receptors. Further studies examining direct or indirect interaction of FSO with purinergic/adenosine receptors at the cell-specific levels and how it affects their functional activity are critical for understanding the upstream implications of this finding. In addition, further studies are needed to explore the potential of FSO in preventing cognitive impairments after intoxication as well as in other neurological conditions.

In summary, FSO strengthened the antioxidant defense system, prevented microgliosis, upregulation of activity, and expression of NTPDase1-eN and purinergic receptors involved in microglial reactivity at the early stage of TMT-induced neurodegeneration. Additionally, it induced alterations in key components of the adenosine system, highlighting potential targets for its neuroprotective activity. Increased expression of A_1_R, A_2B_R, and A_3_R may serve as an adaptive response to enhance neuronal survival, while reduced A_2A_R expression suggests a dampening of inflammatory processes. Downregulation of ENT1 could enhance extracellular adenosine accumulation, while increased expression of ADA likely reflects a potential mechanism to prevent receptor desensitization and maintain functional adenosine signaling. These changes may be attributed to the bioactive compounds of FSO, particularly n-3 PUFAs such as α-linolenic acid, lignans, and/or antioxidants. However, considering the complex composition of FSO, its neuroprotective effects are likely the result of synergistic interactions rather than the action of a single compound. The findings suggest that FSO is more effective than single isolated components and yield to superior effects [3]. As a nutrient that can be consumed daily regardless of disease occurrence, the results suggest that FSO has the potential to modulate the expression of certain components of the purinergic system. These findings may provide further insight into the neurobiological mechanisms underlying the effects of FSO, emphasizing the influence of its dietary intake on brain function.

## 4. Materials and Methods

### 4.1. Animals

The experiments were performed using 10-week-old female rats of the Wistar strain weighing 200–220 g; they were bred at the experimental animal facility of the VINČA Institute of Nuclear Sciences—National Institute of Republic of Serbia, University of Belgrade. Appropriate actions were taken to alleviate the pain and discomfort of the animals in accordance with the European Communities Council Directive (2010/63/EU) for animal experiments, and the research procedures were approved by the Ethical Committee for the Use of Laboratory Animals of VINČA Institute of Nuclear Sciences—National Institute of Republic of Serbia, University of Belgrade, Belgrade, Republic of Serbia (No. 323-07-04787/2019-05). Animals were housed (3–4/cage) under standard conditions: 12 h light/dark regime, constant ambient temperature (22 ± 2 °C), and free access to food and water.

### 4.2. Treatment

Female rats were randomly divided into four experimental groups according to whether they were treated with FSO and/or intoxicated with TMT: a control group without any treatment (Ctrl, *n* = 15), a group with FSO treatment (FSO, *n* = 15), a TMT-intoxicated group without FSO treatment (TMT, *n* = 16), and FSO-treated TMT-intoxicated group (FSO + TMT, *n* = 15), as previously reported [39]. Females in the FSO and FSO + TMT groups were treated daily with flaxseed oil (Granum^®^, Hajdukovo, Serbia, commercial, 1 mL/kg body weight, orally by using a syringe) for two weeks. The fatty acid composition of FSO (Table 1) was confirmed by gas chromatography analysis (Shimadzu 2014, Kyoto, Japan). The dose for FSO was chosen based on data from the literature demonstrating its beneficial effects on various organ systems as well as on the brain [39]. All animals adapted to daily FSO administration without any difficulties. On day 14, a part of the untreated and FSO-treated animals received a single dose of TMT (TMT and FSO + TMT groups), and the application of FSO was continued for both the FSO and FSO + TMT group. The rats were injected i.p. with TMT-chloride (8.0 mg/kg, body weight) dissolved in 0.9% NaCl and then returned to their home cages, as reported [39,42].

We chose an early stage of TMT-induced neurodegeneration because we had previously described that a significant number of damaged neurons were observed as early as 7 days after intoxication and most of the investigated components of the purinergic system were altered at this time-point [39,42,43]. Therefore, the animals were sacrificed 7 days after TMT intoxication and after 3 weeks of FSO treatment.

### 4.3. Hyperactivity/Tremor and Aggression Assessment

TMT triggers specific behavioral patterns that include hyper-excitability, tremors, seizures and aggression [35,36,41,43,44]. Behavior was scored daily by placing freely moving animals in a brightly lit arena (40 × 40 cm, 250 lux) at 5 min intervals using an arbitrarily determined hyperactivity/tremor scale as follows: (0) normal motor activity, (1) hyperactivity and hyper-responsiveness, (2) mild tremor with normal motor activity, (3) systemic tremor [35,43]. The aggression score was measured by the scoring method of Pinel et al. [74]. The scores are as follows: (0) remains calm when approached and grasped, (1) shies from hand when grasped, (2) avoids hand by running, struggles when captured, or both, (3), leaps to avoid capture and struggles vigorously when captured and (4) leaps, struggles, and bites when captured. These symptoms reflect the neurotoxic effects of TMT on the brain, particularly on regions involved in aggression and emotional regulation, such as the amygdala and hippocampus. Upon completing behavioral assessment at day 7 after TMT intoxication, all animals were decapitated using a small animal guillotine (Harvard Apparatus, Holliston, MA, USA), and brains were isolated for tissue processing.

### 4.4. Tissue Processing for Immunohistochemistry and Enzyme Histochemistry

Brains (*n* = 5 animals/group) were immediately removed from the skull, fixed in 4% paraformaldehyde/0.1 M phosphate buffer (pH 7.4) and cryoprotected in graded sucrose solutions (10–30% in 0.2 M phosphate buffer) at 4 °C, as previously reported [39,42,43]. In brief, 20 μm-thick coronal cryostat sections of the dorsal hippocampus were taken starting from level Bregma 2.6 to 3.6 mm, according to the rat stereotaxic atlas of Paxinos and Watson [75].

Immunohistochemistry was performed as previously described [39,42,43]. After washing in phosphate-buffered saline (PBS), which blocked endogenous peroxidase, slides were incubated in 5% donkey normal serum at RT for 1 h to block nonspecific binding. Sections were probed with goat anti-rat ionized calcium-binding adapter molecule-1 (Iba1) antibody (1:500 dilution, Abcam, Cambridge, UK, ab5076) overnight at 4 °C. After washing in PBS, sections were incubated with goat anti-mouse HRP-conjugated secondary antibody (1:150 dilutions, R&D Systems, Minneapolis, MN, USA, HAF007). The signal was visualized with the use of 3, 3′-S-diaminobenzidine-tetrahydrochloride kit (DAB, Abcam, Cambridge, UK) as a chromogen for HRP-conjugated secondary antibodies. The sections were dehydrated in graded ethanol (70, 95, and 100% EtOH, and 100% xylol) and mounted with DPX medium (Sigma-Aldrich, St. Louis, MO, USA).

Enzyme histochemistry using ADP and AMP as a substrate was performed as previously described [42,45]. Cryosections were preincubated for 30 min at room temperature with TRIS-maleate sucrose (TMS) buffer containing 0.25 M sucrose, 50 mM TRIS-maleate, 2 mM levamisole, and 2 mM MgCl_2_ (pH 7.4). The enzyme reaction was performed at 37 °C in a TMS-buffered substrate solution for 60 min, containing 1 mM ADP or AMP, in addition to 2 mM Pb(NO_3_)_2_, 5 mM MnCl_2_, 2 mM MgCl_2_, 50 mM TRIS-maleate, 3% dextran T250, and 0.25 M sucrose (pH 7.4). TMS-buffered substrate solution without substrate served as a control. After thorough washing and subsequent color development (1% (*v*/*v*) (NH_4_)_2_S), a brown deposit of the enzyme reaction product became visible. The sections were dehydrated in graded ethanol (70, 95, and 100% EtOH, and 100% xylol) and mounted with DPX medium (Sigma-Aldrich, St. Louis, MO, USA). Application of alkaline phosphatase inhibitor levamisole produced no significant effect on the histochemical staining.

The digital images were acquired using LEITZ DM RB light microscope (Leica Mikroskopie & Systems GmbH, Wetzlar, Germany), an LEICA DFC320 CCD camera (Leica Microsystems Ltd., Heerbrugg, Switzerland), and LEICA DFC Twain Software v. 7.3 (Leica Mikroskopie & Systems, Germany).

### 4.5. Gene Expression Analysis by RT-qPCR

Total RNA from the whole hippocampal formations (*n* = 5–6 animals/group) were extracted using TRIzol Reagent (Invitrogen, Waltham, MA, USA) according to the manufacturer’s protocol as previously described [39,42]. The concentration and the purity of RNA were determined using the OD260/OD230 and OD260/OD280 ratios, respectively. Complementary DNA (cDNA) species were synthesized using a High-Capacity cDNA Reverse Transcription Kit (Thermo Fisher Scientific, Waltham, MA, USA), as previously described [39,42]. Quantitative real-time PCR was performed using Power SYBR™ Green PCR Master Mix (Applied Biosystems, Waltham, MA, USA), and an ABI Prism 7000 Sequence Detection System (Applied Biosystems, Waltham, MA, USA). The primer sequences used for amplification are listed in Table 2. To ensure accurate normalization of gene expression data, we initially evaluated three commonly used housekeeping genes: glyceraldehyde-3-phosphate dehydrogenase (*Gapdh*), cyclophilin A (*CycA*), and hypoxanthine-guanine phosphoribosyltransferase (*Hprt*). The cycle threshold (Ct) values were within the same half of the cycle for *CycA* and *Hprt* in all samples examined, so they could be accepted as reference genes. Based on this validation, we selected *CycA* for final normalization using the 2^−ΔΔCT^ method, as it had the most stable expression in our samples. All samples were run in duplicate and internal standard curves were generated in each run by multiple dilutions of the generated cDNA to check for amplification efficiency. At the end of each experiment, a melting curve analysis was performed to confirm the formation of a single PCR product.

### 4.6. Preparation of Membrane Fraction

After decapitation, brains (5 per group) were isolated for preparations of crude membrane (P2) fractions that contained a bulk of synaptosomes and other membrane fragments, such as glial cells and neuronal membranes, as described previously [39,76,77]. Hippocampi were suspended in 10 volumes of ice-cold medium (0.32 M sucrose, 5 mM HEPES, pH 7.4) and homogenized in a Teflon/glass homogenizer (clearance 0.20 mm) at 900 rpm. A crude nuclear fraction and cell debris were removed by centrifugation at 1000× *g* for 10 min. Supernatants were collected and centrifuged at 16,000× *g* for 30 min to obtain a P2 fraction, which was resuspended in 5 mM HEPES, pH 7.4. All steps were carried out at 4 °C. The samples were aliquoted and stored at −80 °C until use. The P2 fractions were separately isolated from individual animals without pooling the tissue. The protein content was determined using bovine serum albumin as a standard.

### 4.7. SDS-PAGE and Immunoblotting

SDS-PAGE and immunoblotting were performed as previously described [39,76,77]. Briefly, equivalent amounts (20 μg of total proteins) were resolved by SDS-PAGE (4–8%) and transferred onto PVDF support membranes (0.45 mm, Millipore, Darmstadt, Germany). After washing in TBST (50 mM Tris-HCl pH 7.4, 150 mM NaCl, 0.05% Tween 20), the membranes were blocked in 5% Blotto (Santa Cruz Biotechnology, Inc., Dallas, Texas, USA) in TBST for 1 h. The membranes were incubated with guinea pig anti-rat NTPDase1 antibodies (1:1000 dilution; ab66215, Abcam, Cambridge, UK), rabbit anti-rat CD73 (1:2000 dilution; mAb#13160, Cell Signaling Technology, Danvers, MA, USA), rabbit anti-rat ADA (1:1000 dilution: E-AB-66162, Elabsciences, Houston, TX, USA), and rabbit anti-rat ENT1 (1:1000 dilution; 29862-1-AP, Proteintech, Planegg-Martinsried, Germany) overnight at 4 °C. After washing with TBST, membranes were incubated with an appropriate horseradish–peroxidase-conjugated secondary antibody (1:10,000 dilutions in TBST) for 1 h at room temperature. Support membranes were re-probed with anti-β actin antibody HRP-conjugated (1:5000 dilution; ab49900, Abcam, Cambridge, UK) after visualization of the target protein. The visualization of the specific bands was performed on an iBright CL1500 Imaging System (Thermo Fischer Scientific, Waltham, MA, USA) with a commercial chemiluminescence kit (Immobilon Western Chemiluminescent HRP substrate, Millipore, Darmstadt, Germany). A densitometric analysis was performed using the ImageJ 1.53t software package, and the optical density of each band was normalized by the optical density of the β-actin band from the same lane. Results are presented as mean protein abundance relative to β-actin ± SD, from 5 individual samples/group each run in technical triplicates.

### 4.8. Ectonucleotidase Assays

The NTPDase and eN activities were evaluated by measuring the formation of inorganic phosphate (Pi) upon addition of ATP, ADP, or AMP to hippocampal membrane P2 preparations, as described previously [76,77]. Briefly, an aliquot of P2 fraction containing 10 μg of total proteins was resuspended in assay buffer, preincubated for 10 min at 37 °C, and incubated with the 1 mM ATP or ADP for 10 min, or 1 mM AMP for 30 min, at 37 °C. The levels of Pi liberated by enzyme activities were determined by the Malachite green method. The optical density was estimated at 650 nm using 96-well plates in a microplate reader (Wallac 1420 VICTOR, Perkin Elmer Instruments, Waltham, MA, USA). The conditions for the enzyme analyses were chosen in separate experiments in order to ensure linearity of the reactions. The separate measurements from five independent preparations/group were run in triplicate, and the activity of each sample was expressed as mean specific activity ± SD (in nmol Pi/mg protein/min).

### 4.9. Adenosine Deaminase (ADA) Assay

ADA catalyzes the deamination of adenosine to inosine with the release of ammonium ions, which are used for the spectrophotometric measurements of ADA enzyme activity, as reported [28,76]. Briefly, 50 µg in HEPES (pH 7.4) of total proteins were preincubated for 10 min at 37 °C. Adenosine was added in a final concentration of 1.5 mM and the samples were incubated for 2 h at 37 °C. Reaction mixture without adenosine was used as a adenosine-free blank control. The reaction was stopped by sequential addition of 500 µL of Reagent I (68 g/L salicylic acid, 25 g/L sodium hydroxide, and 2.2 g/L sodium nitroprusside) and 500 µL of Reagent II (40.9 mL/L sodium hypochlorite), and the resulting diazonium salt was spectrophotometrically detected at 620 nm after 15 min. The amount of ammonium ions released in the reaction was calculated from the standard curve, with NH_4_Cl used as a standard. The results were presented as mean specific activity (nmol NH_4_^+^/mg protein/h) ± SD, from 5 independent preparations/group, run in triplicate.

### 4.10. Data Analysis

Data were analyzed for normality and appropriate parametric tests were used. All values are presented as mean ± SD of independent determination. For multiple comparisons, we used two-way ANOVA followed by Tuckey’s post hoc multiple comparison test. The values of *p* < 0.05 or less and a target power of 80–95% were considered statistically significant. Data were analyzed using the Prism—GraphPad 6.07 software package.

## Figures and Tables

**Table 1:** Fatty acid composition of FSO.

Fatty Acids	%
C16:0	7.96 ± 0.52
C16:1	0.18 ± 0.04
C18:0	4.29 ± 0.26
C18:1 (n-9)	22.52 ± 0.88
C18:1 (n-7)	0.00 ± 0.00
C18:2	25.34 ± 3.10
C18:3 (n-6)	0.32 ± 0.09
C18:3 (n-3)	39.16 ± 1.32
C20:3	0.22 ± 0.08
C20:4	0.00 ± 0.00
C20:5	0.00 ± 0.00
C22:4	0.00 ± 0.00
C22:5	0.00 ± 0.00
C22:6	0.00 ± 0.00
SFA *	12.25 ± 0.77
MUFA *	22.71 ± 0.84
PUFA *	65.04 ± 1.61

* SFA, saturated fatty acids; MUFA, monounsaturated fatty acid; PUFA, polyunsaturated fatty acids.

**Table 2:** Primer sequences used for RT-qPCR.

Gene	Sequence (5′-3′)	Length (bp)
**Nrf2 (*Nfe2l2*)**	GACTTGGAATTGCCACCGC CCTGTTCCTTCTGGAGTTGCT	193
**NF-kB (*Rela*)**	AGCATGTACAGATTCTGGGGAG AGAGCCGACTATCGTACAGGG	195
**GPx1 (*Gpx1*)**	AGCGACCAGATGAAGCAGTG TCCGCTCTCTGTCAAAGTGTG	181
**Gsr (*Gsr*)**	CCCACATCGAAGTCATCCAC GATCAGGATGTGTGGAGCAG	101
**SOD2 (*Sod2*)**	TGACCTGCCTTACGACTATGG CTCGTGGTACTTCTCCTCGG	127
**CAT (*Cat*)**	TAATATCATGACTGCGGGGC TCTCTCAGGAATCCGCTCTC	100
**NTPDase1** ***(Entpd1)***	TCAAGGACCCGTGCTTTTAC TCTGGTGGCACTGTTCGTAG	150
**eN *(Nt5e)***	CAAATCTGCCTCTGGAAAGC ACCTTCCAGAAGGACCCTGT	160
**P2X_4_R *(P2rx4)***	ACCAGGAAACGGACTCTGTG TCACGGTGACGATCATGTTGG	168
**P2Y_6_R *(P2ry6)***	CAGTTATGGAGCGGGACAAT GTAAACTGGGGGTAGCAGCA	104
**P2Y_12_R *(P2ry12)***	TCACCCGCACCCTCTATTAC GCCAGGAAGTAGAGCACAGG	139
**A1R (*Adora1)***	GTGATTTGGGCTGTGAAGGT GAGCTCTGGGTGAGGATGAG	194
**A_2A_R (*Adora2a*)**	TGCAGAACGTCACCAACTTC CAAAACAGGCGAAGAAGAGG	141
**A_2B_R *(Adora2b)***	CGTCCCGCTCAGGTATAAAG CCAGGAAAGGAGTCAGTCCA	104
**A_3_R *(Adora3)***	TTCTTGTTTGCCTTGTGCTG AGGGTTCATCATGGAGTTCG	129
**NGF *(Ngf*)**	CTGGAGCCGAAGGGGAG ACTGAGGTGAGCTTGGGTCC	103
**ADA *(Ada)***	GAGCCTCATCCTGTGAATGG ATGCCCATGATTGTCAAGGT	143
**ENT1 (*Slc29a1*)**	CACTTCCTTCGCTGTTAGGG TGTCCCCCTACCACTCTGAC	144
**CycA (*Ppia*)**	GGCAAATGCTGGACCAAACAC TTAGAGTTGTCCACAGTCGGAGATG	196

## Data Availability

The data that support the findings of this study are available from the corresponding author upon reasonable request.

## Supplementary Materials

The following supporting information can be downloaded at https://www.mdpi.com/article/10.3390/ijms262110283/s1.

## Abbreviations

ADP	adenosine diphosphate
ATP	adenosine triphosphate
AMP	adenosine monophosphate
Cat	catalase
eN	ecto-5’-nucleotidase
ENT1s	equilibrative nucleoside transporter 1
FSO	flaxseed oil
Gpx1	glutathione peroxidase 1
Gsr	glutathione reductase
n-3 PUFA	omega-3 polyunsaturated fatty acids
NF-κB	nuclear factor kappa-light-chain-enhancer of activated B cells
NGF	nerve growth factor
Nrf2	nuclear factor erythroid 2-related factor 2
NTPDase1	ecto-nucleoside triphosphate diphosphohydrolase 1
Sod2	superoxide dismutase 2
TMT	trimethyltin
