# Supplementary material for: Neuroprotection by Flaxseed Oil in a Model of Hippocampal Injury Induced by Trimethyltin Involves Purinergic System Modulation
Source: Int J Mol Sci. 2025 Oct 22;26(21):10283. doi: 10.3390/ijms262110283 (PMC12609297; doi:10.3390/ijms262110283)
Supplement: Supplementary file 1 [file ijms-26-10283-s001.zip › ijms-3920895-supplementary.pdf]

Table S1. Results of two-way ANOVA for qPCR analysis.

| **Two-way ANOVA** | | | |
|---|---|---|---|
| **Target mRNA** | **FSO** | **TMT** | **FSO x TMT** |
| **NRF2** | F (1, 20) = 34.03, P < 0.0001 | F (1, 20) = 1.532, P = 0.2302; **NS** | F (1, 20) = 26.38, P < 0.0001 |
| **NF-kB** | F (1, 20) = 11.81, P = 0.0026 | F (1, 20) = 4.110, P = 0.0562; **NS** | F (1, 20) = 14.27, P = 0.0012 |
| **Gpx1** | F (1, 20) = 3.050, P = 0.0961; **NS** | F (1, 20) = 5.073, P = 0.0357 | F (1, 20) = 11.87, P = 0.0026 |
| **Gsr** | F (1, 20) = 6.278, P = 0.0210 | F (1, 20) = 0.04026, P = 0.8430; **NS** | F (1, 20) = 0.4137, P = 0.5274; **NS** |
| **Sod2** | F (1, 20) = 4.617, P = 0.0441 | F (1, 20) = 1.757, P = 0.1999; **NS** | F (1, 20) = 0.1466, P = 0.7058; **NS** |
| **Cat** | F (1, 20) = 32.26, P < 0.0001 | F (1, 20) = 0.4688, P = 0.5014; **NS** | F (1, 20) = 0.1547, P = 0.6982; **NS** |
| **CD39** | F (1, 16) = 31.02, P < 0.0001 | F (1, 16) = 21.89, P = 0.0003 | F (1, 16) = 0.001476, P = 0.9698; **NS** |
| **CD73** | F (1, 16) = 12.01, P = 0.0032 | F (1, 16) = 3.802, P = 0.069; **NS** | F (1, 16) = 0.6751, P = 0.4246; **NS** |
| **P2X$_4$R** | F (1, 16) = 15.34, P = 0.0012 | F (1, 16) = 36.87, P < 0.0001 | F (1, 16) = 36.70, P < 0.0001 |
| **P2Y$_6$R** | F (1, 17) = 5.019, P = 0.0387 | F (1, 17) = 35.64, P < 0.0001 | F (1, 17) = 22.57, P = 0.0002 |
| **P2Y$_{12}$R** | F (1, 17) = 24.72, P = 0.0001 | F (1, 17) = 35.50, P < 0.0001 | F (1, 17) = 25.59, P < 0.0001 |
| **A$_1$R** | F (1, 16) = 76.88, P < 0.0001 | F (1, 16) = 1.695, P = 0.2114; **NS** | F (1, 16) = 1.236, P = 0.2827; **NS** |
| **A$_{2A}$R** | F (1, 16) = 52.05, P < 0.0001 | F (1, 16) = 1.225, P = 0.2848; **NS** | F (1, 16) = 4.392, P = 0.0524; **NS** |
| **A$_{2B}$R** | F (1, 16) = 40.55, P < 0.0001 | F (1, 16) = 3.017, P = 0.1016; **NS** | F (1, 16) = 1.082, P = 0.3137; **NS** |
| **A$_3$R** | F (1, 16) = 4.390, P = 0.0524; **NS** | F (1, 16) = 1.065, P = 0.3174; **NS** | F (1, 16) = 4.676, P = 0.0461 |
| **NGF** | F (1, 16) = 109.4, P < 0.0001 | F (1, 16) = 25.38, P = 0.0001 | F (1, 16) = 2.744, P = 0.1171; **NS** |
| **ADA** | F (1, 16) = 0.1042, P = 0.7510 **NS** | F (1, 16) = 30.01 P = 0.0001; | F (1, 16) = 2.028, P = 0.1736; **NS** |
| **ENT1** | F (1, 16) = 85.49, P < 0.0001 | F (1, 16) = 14.00, P = 0.0018 | F (1, 16) = 4.482, P = 0.0503; **NS** |

Table S2. Results of two-way ANOVA for Western blott analysis.

| Two-way ANOVA | | | |
|---|---|---|---|
| **Target protein** | **FSO** | **TMT** | **FSO x TMT** |
| **NTPDase1/CD39** | F (1, 16) = 7.810, P = 0.0130 | F (1, 16) = 12.56, P = 0.0027 | F (1, 16) = 4.535, P = 0.0491 |
| **eN/CD73** | F (1, 16) = 0.5121, P = 0.4845; **NS** | F (1, 16) = 12.34, P = 0.0029 | F (1, 16) = 5.872, P = 0.0276 |
| **ADA** | F (1, 16) = 23,15, P = 0,0002 | F (1, 16) = 0,1425, P = 0,7107; **NS** | F (1, 16) = 6,691, P = 0,0199 |
| **ENT1** | F (1, 16) = 5,899, P = 0,0273 | F (1, 16) = 6,266, P = 0,0235 | F (1, 16) = 3,459, P = 0,0814; **NS** |

Table S3. Results of two-way ANOVA for enzyme activity assay.

| Two-way ANOVA | | | |
|---|---|---|---|
| **Enzyme assay** | **FSO** | **TMT** | **FSO x TMT** |
| **ATP hydrolysis** | F (1, 16) = 5.850, P = 0.0279 | F (1, 16) = 32.34, P < 0.0001 | F (1, 16) = 10.01, P = 0.0060 |
| **ADP hydrolysis** | F (1, 16) = 12.07, P = 0.0031 | F (1, 16) = 23.27, P = 0.0002 | F (1, 16) = 20.22, P = 0.0004 |
| **AMP hydrolysis** | F (1, 16) = 2.443, P = 0.1376; **NS** | F (1, 16) = 10.19, P = 0.0057 | F (1, 16) = 7.491, P = 0.0146 |
| **ADA hydrolysis** | F (1, 16) = 21.30, P = 0.0003 | F (1, 16) = 0.3960, P = 0.5380; **NS** | F (1, 16) = 4.659, P = 0.0464 |
